# Interaction between three key species in the sea ice-reduced Arctic Barents Sea system

**DOI:** 10.1098/rspb.2024.1408

**Published:** 2024-10-09

**Authors:** Joël M. Durant, Nicolas Dupont, Kotaro Ono, Øystein Langangen

**Affiliations:** ^1^ Department of Biosciences, Centre for Ecological and Evolutionary Synthesis (CEES), University of Oslo, PO Box 1066, Blindern, Oslo NO-0316, Norway; ^2^ Institute for Marine Research (IMR), Bergen, Norway; ^3^ Department of Biosciences, Section for Aquatic Biology and Toxicology (AQUA), University of Oslo, PO Box 1066, Blindern, Oslo NO-0316, Norway

**Keywords:** climate change, scenario testing, Arctic, trophic interaction, fish

## Abstract

Population dynamics depend on trophic interactions that are affected by climate change. The rise in sea temperature is associated with the disappearance of sea ice in the Arctic. In the Arctic part of the Barents Sea, Atlantic cod, capelin and polar cod are three fish populations that interact and are confronted with climate-induced sea ice reductions. The first is a major predator in the system, while the last two are key species in Arctic and sub-Arctic ecosystems, respectively. There are still many unknowns regarding how predicted environmental change may influence the joint dynamics of these populations. Using time series from a 32 year long survey, we developed a state-space model that jointly modelled the dynamics of cod, capelin and polar cod. Using a hindcast scenario approach, we projected the effect of reduced sea ice on these populations. We show that the impact of sea ice reduction and concomitant sea temperature increase may lead to a decrease of polar cod abundance at the benefit of capelin but not of cod which may decrease, resulting in strong changes in the food web. Our analyses show that climate change in the Arcto-boreal system can generate different species assemblages and new trophic interactions, which is the knowledge needed for effective management measures.

## 1. Introduction

The Arctic experiences dramatic changes related to climate change. The most salient change is the disappearance of the sea ice [1], which is linked to the warming of sea temperature and reduced sea ice import [2]. For instance, the Barents Sea is a productive sea adjacent to the Arctic Ocean that is historically seasonally covered by sea ice and sustains a boreal ecosystem in the southwest and an Arctic marine ecosystem in the northeast. Linked to climate change, sea ice cover in the Barents Sea has been steadily decreasing for all months of the year [3], and this trend is expected to continue in the future [1]. A consequence of these physical changes is a ‘borealization’ [4] of the northern parts of the Barents Sea. The term refers to the expansion of boreal species northwards, where they interact with Arctic species and may establish new trophic interactions [5–7] as observed in other places in the Arctic [8]. For instance, the Barents Sea hosts large and economically important boreal fish populations, such as the northeast Arctic (NEA) cod (*Gadus morhua*) and the Barents Sea capelin (*Mallotus villosus*) [9]. The piscivorous NEA cod and planktivorous capelin are expanding north-eastward into areas previously occupied by Arctic fish species such as polar cod (*Boreogadus saida*) [4,10]. Consequently, the Arctic species are experiencing increased competition and predation pressure from incoming boreal species [11]. The reduction in sea ice also directly affects ice-associated species such as polar cod that inhabit the Arctic area [12,13]. For example, sea ice is important as a refuge for young polar cod as it is for the biomass or phenology of its prey populations [13,14]. The NEA cod is an ecologically important generalist predator with the potential capacity to shape the prey community and ecosystem functioning through predation [15–17]. Polar cod and capelin are abundant key species, respectively, in Arctic and sub-Arctic ecosystems [14] with partially overlapping distributions, where they may compete for the same zooplankton resources [18]. With the ‘borealization’ of the Barents Sea, the overlap and interaction of these three fish species are increasing [10]. Management bodies consider the interaction between NEA cod, capelin and polar cod as a key factor regulating these population abundances [14,19]. The Barents Sea capelin is considered to be the main food for NEA cod [20,21], and low capelin stock was considered one of the reasons for the very low NEA cod catches at the end of the 1980s [22]. In turn, predation by NEA cod on capelin, in association with increased fishing pressure and reduced food availability, is thought to have delayed the capelin stock’s recovery after its collapses [22]. It has also been suggested that an increase in the consumption of polar cod by NEA cod would be associated with low stock biomass of capelin [18]. In addition to predation, climate change may increase food competition between polar cod and capelin [18] but also with the young Norwegian spring spawning herring (*Clupea harengus*) growing in the Barents Sea [13,23]. Young herrings have also been shown to negatively affect the NEA cod in the Barents Sea since the 1980s [24]. Finally, NEA cod, capelin and polar cod population dynamics are well documented to be affected by environmental variables [12,25,26], such as temperature that influences metabolic processes and growth rates in fish [27,28].

However, we still have limited knowledge on how abiotic environmental changes in the Arctic, i.e. climate change and resulting sea ice reduction, will affect the interplay between these three species. We hypothesize that polar cod will be negatively affected by a sea ice reduction due to an increased predation by NEA cod and competition by capelin, while the last two will benefit from an increased sea temperature.

With the ‘borealization’ of the Barents Sea, the overlap and interaction of ecological and economically important Barents Sea capelin, polar cod and NEA cod populations are increasing. Here, we applied a Gompertz state-space model [29–31] on a 32 year long survey time series of these three species aiming to: (i) assess whether there are population-level effects of the known interaction between the three species and (ii) understand how the reduction of sea ice extent in the Barents Sea due to climate change will affect the interaction of these three species.

## 2. Methods

### (a) Data

**Figure 1.**
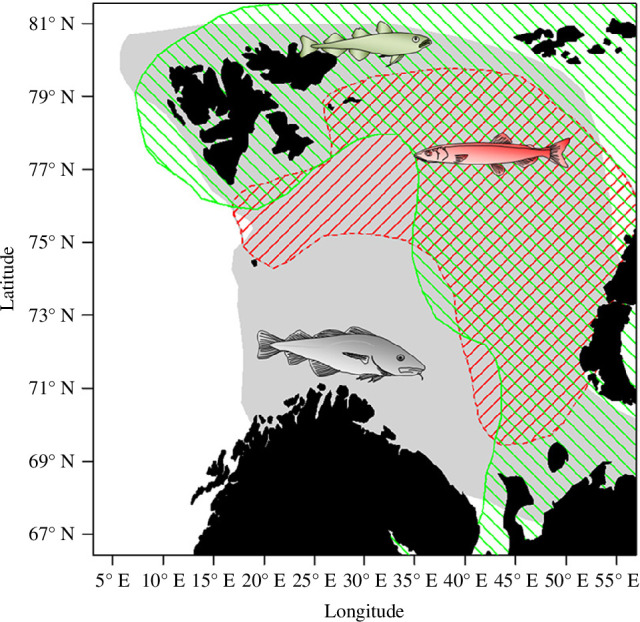
Approximate feeding distributions in the Barents Sea of northeast Arctic cod (grey), capelin (red) and polar cod (green). The map is redrawn from [32].

We jointly analysed the change in population abundance for three fish populations in the Barents Sea: polar cod, Barents Sea capelin and NEA cod (figure 1). Population total abundance data (1986–2017) were extracted from reports: Capelin (*Cap*) abundances (in numbers 10^9^) are estimates from the August to September acoustic survey (table 9.4, [33]); NEA cod (*Cod*) abundances (in numbers 10^6^) are acoustic estimates from the joint winter Barents Sea survey and the Norwegian Lofoten acoustic survey (table A13, [33]); Polar cod (*Pcod*) abundance are derived from juvenile and adult abundance (in numbers 10^9^) acoustic estimates in autumn (table 7.2.2.2, [34]) and 0-group abundance (table 6.1, [35]).

In addition, we used data of potential predators and food competitors: the Norwegian spring spawning herring *C. harengus* (10^6^ tonnes, February to May, *Herring*, [36]).

We also used two climatic variables (the Kola transect sea temperature (ST) and the sea ice index (Ice)) as potential environmental drivers of population dynamics [29]. The sea temperature (1921–2019) is an aggregated yearly average (in °C) over the upper 200 m at five stations (3–7) on the Kola meridian transect (33°30′ E and 70°30′−72°30′ N) in the Barents Sea (http://www.pinro.vniro.ru/). The sea ice index is a climate indicator of sea ice coverage for the Barents Sea (in 10^5^ km^2^). The sea ice index is derived from the Ocean and Sea Ice Satellite Application Facility (OSI SAF) Global Sea Ice Concentration data (https://osi-saf.eumetsat.int/products/osi-420). We calculated the mean sea ice index (thereafter Ice) for the winter (December to May), the 6 months with the highest mean sea ice index value. Finally, we took into account the fishing pressure that is particularly relevant for cod by including the fishing mortality index *F* (table 3.18, [37]) for NEA cod and by calculating a proxy for the fishing mortality by using the formula −ln(1 − Landing/Biomass) using data from (table 10.5, [37]) for capelin, as well as landings data (https://www.ices.dk/data/dataset-collections/Pages/Fish-catch-and-stock-assessment.aspx) and biomasses (table 7.2.2.2, [34]) for polar cod. Both environmental variables and *F* are *z*-score transformed, thus making the estimated parameters easier to compare by bringing the predictor variables to the same scale.

### (b) Model description

The analysis was based on a Gompertz state-space model adapted to predict fish population dynamics [29–31,38,39]. The model incorporated a Gaussian-distributed stochastic term (*ε*) to acknowledge our inadequate understanding of the population *i* dynamics complexity (i.e. the process error, see later).


$$ln(Cod_{yr})=a_{cod,0}+a_{cod,cod}\cdot ln(Cod_{yr-1})+a_{cod,cap}\cdot ln(Cap_{yr-1})+a_{cod,pcod}\cdot ln(Pcod_{yr-1})+a_{cod,herring}\cdot ln(Herring_{yr-1})\;\;\;\;+a_{cod,st}\cdot ST_{yr-1}+a_{cod,F}\cdot F_{yr-1}+\varepsilon_{cod,yr}\;ln(Cap_{yr})=a_{cap,0}+a_{cap,cap}\cdot ln(Cap_{yr-1})+a_{cap,pcod}\cdot ln(Pcod_{yr-1})+a_{cap,cod}\cdot ln(Cod_{yr})+a_{cap,herring}\cdot ln(Herring_{yr})\;\;\;\;+a_{cap,st}\cdot ST_{yr-1}+a_{cap,F}\cdot F_{yr-1}+\varepsilon_{cod,yr}\;ln(Pcop_{yr})=a_{pcod,0}+a_{pcod,pcod}\cdot ln(Pcod_{yr-1})+a_{pcod,cap}\cdot ln(Cap_{yr-1})+a_{pcod,cod}\cdot ln(Cod_{yr})+a_{pcod,herring}\cdot ln(Herring_{yr})\;\;\;\;+a_{pcod,ice}\cdot Ice_{yr}+ap_{cod,F}\cdot F_{yr-1}+\varepsilon_{pcod,yr}\;$$

Note that cod and herring surveys at year yr, as well as the mean winter sea ice index (Ice), are conducted between the capelin and polar cod surveys at year yr−1 and year yr. Consequently, for these variables, we used estimates at year yr (i.e. Cod_yr_, Herring_yr_ and Ice_yr_) instead of at year yr−1 when modelling capelin and polar cod. A summary of the relationships considered that explain the model formulation is given in electronic supplementary material, figure S1. Note that ST and Ice variables are highly correlated (*r* = −0.75, *p* < 0.01; see §2c) and that ST measured on the Kola meridian transect is of limited relevance for the polar cod. We used only one of each per model: Ice for the polar cod model and ST for the two others.

The process errors for the three species models (ε_cod,yr_, ε_cap,yr_ and ε_pcod,yr_) were jointly drawn from a multivariate normal distribution to account for correlated process errors between the three species. In biological terms, the process errors represent, e.g. the influence of unmeasured factors that may affect the population dynamics of the studied species.

We assumed that the observed abundances (obs; from survey) were normally distributed (in log scale) with variance term *σ*^2^_*i*,obs_ around the true log population values for the species *i*.

We used Hamiltonian Monte Carlo (HMC) through the Stan software via the R package *rstan* (v.2.21.3) to simulate from the joint posterior distribution of the model parameters (for cod, capelin and polar cod) in a single model for the period 1986–2017 [40]. We used *shinystan* for visual inspection and convergence diagnostics [40]. Weakly informative priors were used to let the data drive the inferences except for the process and observation error variances. The observation error variances were not estimable with the data we had, and we included an informative prior on the ratio of the process to observation error variance centred around 2 (*σ*^2^_*i*,obs_ = Normal(2, 0.5) × *σ*^2^_*i*,proc_) for each species *i* [41]. A sensitivity test with a normally distributed random ratio centred around 1 and 3 (respectively, Normal(1, 0.5) and Normal(3, 0.5)) showed that the choice of the exact value of the ratio did not affect our results much. To account for the unrealistically low polar cod abundance in 1995, we added a specific observation error term for this year in the polar cod observation model [12]. The prior values are reported in electronic supplementary material, table S1, and specifications in the model description in the programming language in the electronic supplementary material.

We used four independent chains with 30 000 iterations each, where the first 20 000 iterations were discarded as ‘burn-in’ iterations. In addition, we thinned the chains with a factor of 10 to reduce autocorrelation in the posterior samples and to produce a reasonable amount of output (*n* = 4000). We used the Ȓ convergence diagnostics as implemented in *rstan* [42] and visual inspection of the chains to ensure convergence, and posterior predictive checks to evaluate the model fit. All analyses were conducted using the software R v.4.1.3 [43].

### (c) Hindcast scenario

To explore the effect of environmental conditions on the three populations, we used a hindcast approach that simulated the dynamics of past environmental values under a scenario representing a different climate state (as represented by sea ice and sea temperature). First, we extracted the process errors (ε_cod,yr_, ε_cap,yr_ and ε_pcod,yr_) estimated at each time step and species (4000 MCMC samples per year per species) from the original model fitted to the data. Second, using the posterior samples of the model parameters (excluding the process errors), we simulated a new deterministic time series of abundances for NEA cod, capelin and polar cod following the values of the scenario-specific explanatory variables. This means that we used new values in the equations for sea ice and sea temperature representing the new climate state while keeping the observed values for the remaining variables (herring and fishing). Third, the extracted process errors from step 1 were added back to the simulated abundance values to create the hindcast scenario time series. Finally, we kept only iterations that lead to a stationary time series [39]. Two hundred and thirty-seven iterations (of the 4000) were thus removed from the scenario testing.

We evaluated a low sea ice hindcast scenario following the sea ice extent prediction for the next decades in the Barents Sea [1]. Hindcast scenario data were simulated while keeping the temporal autocorrelation pattern in the original sea ice data. To do this, we estimated the linear relationship between the mean winter sea ice index and year (ln(Ice) = 128.3733 ± 28.2311 (standard error (SE)) − 0.0642 ± 0.0141 (SE) × year, *p* < 0.001, *R*^2^ = 0.41) and extracted the residuals. The new Ice values for the low sea ice hindcast scenario were then calculated using a new relationship between years and ln(Ice) with a reduced slope (−0.005, slope of the line going from the recorded ln(Ice) in 1986 to the one estimated by the first linear relationship for 2017) and intercept (8.316, calculated for the new relationship to pass through the recorded ln(Ice) for 2017). For this new linear relationship, we added the residuals from the first linear relationship to keep the same yearly variation pattern. However, before adding them, the residuals were reduced by 30% to get a new sea ice extent time series with values always lower than the 50% observed extent. Finally, we kept the first measured sea ice value as the starting value (see details in electronic supplementary material, figure S2A).

Since sea ice extent is linearly correlated to sea temperature (ST = −0.7498 ± 0.1210 (SE) × Ice + 0.0455 ± 0.1126 (SE), *p* < 0.001, *R*^2^ = 0.56), we also used new ST following the new sea ice data in our hindcast scenario (electronic supplementary material, figure S2B). Finally, for each year of the time series, we performed 100 bootstraps estimating ST from the ice value to take into account the variability around the linear relationship between ST and Ice (i.e. the SE) and retained the medians to get only one ST value per year.

## 3. Results

On visual inspection, the four parallel Monte Carlo chains were well mixed, had no divergent transitions, had low autocorrelation after thinning and displayed no trends after the burn-in iterations. The Ȓ convergence diagnostics were less than 1.001 for all model parameters, suggesting the model reached convergence. In addition, there was no systematic deviation between the fitted values and the observed time series (electronic supplementary material, figure S3), suggesting the model was well formulated. Our models described the data well (electronic supplementary material, figure S3) with a median Bayesian *R*-square over 80% for NEA cod and capelin and over 60% for polar cod (electronic supplementary material, figure S4). The importance of the process error remained reasonably low indicating that we have accounted for major explanatory variables using an adequate model formulation.

**Figure 2.**
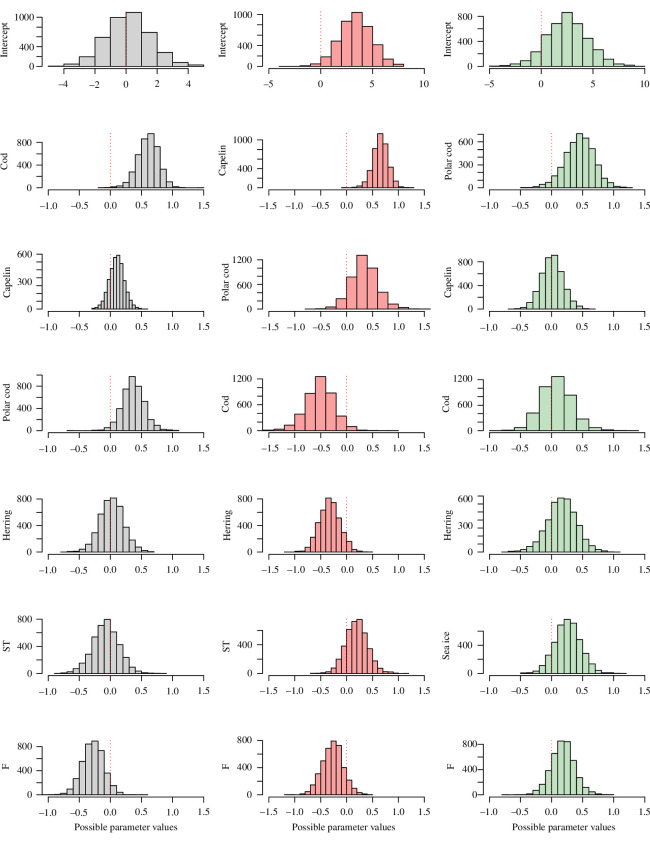
Histograms of the posteriors distribution of the models’ parameters with the zero line (red dotted) indicated. The histograms for the NEA cod model (grey), the histograms for the capelin model (red) and the histograms for the polar cod model (green).

**Figure 3.**
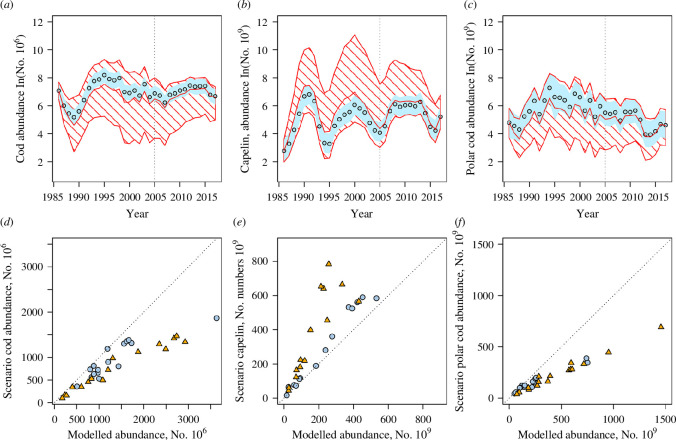
Abundance estimates (upper row) and comparison of median abundances (lower row) between model and hindcast scenario output for NEA cod (plots *a* and *d*), capelin (plots *b* and *e*) and polar cod (plots *c* and *f*). On the upper row, plots are presented in blue, the median (dots) and the unperturbed models’ 95% confidence interval (blue area). The red lines and the dashed areas are the median and the 95% confidence interval for the hindcast scenario. The vertical dotted lines indicate the year 2005, before which most sea ice was abundant and most affected by the scenario (electronic supplementary material, figure S5). For the lower row, the dotted line corresponds to the slope = 1 (no change between the hindcast scenario and the unperturbed model). The orange triangles correspond to the years when sea ice was most changed by the hindcast scenario (over 50% quantile, before *ca* 2005), and the blue dots represent years when sea ice was only marginally different from the hindcast scenario values. Note that the *x*-axis and the *y*-axis are not at the same scale.

Median parameter estimates from these models are presented in table 1, full marginal posterior distributions in figure 2 and the median predicted abundance using the model with confidence interval in figure 3*a*–*c*. The estimates show a strong positive effect of the intraspecific term for all species. They show a negative effect of fishing (F) for NEA cod and capelin but a positive effect for polar cod (indicating that they follow each other but may not affect each other, fishing having been reduced for this species).

**Table 1.** Estimated parameters for the three models. Subscripts ‘cod’ stands for northeast Arctic cod, ‘cap’ stands for Barents Sea capelin and ‘pcod’ stands for polar cod. For comparison purposes, we have scaled the regression coefficients (effect strength) by dividing each explanatory variable parameter median by its standard deviation (not done for st, *F* and ice that were *z*-scored).

species	parameter	median	5%	95%	effect strength
NEA cod	a_cod,0_	0.14	−2.42	3.07	1.17
	a_cod,cod_	0.61	0.28	0.92	5.08
	a_cod,cap_	0.10	−0.12	0.35	0.41
	a_cod,pcod_	0.37	0.03	0.72	1.71
	a_cod,herring_	0.02	−0.36	0.40	0.03
	a_cod,st_	−0.07	−0.49	0.35	−0.07
	a_cod,F_	−0.28	−0.61	0.07	−0.28
capelin	a_cap,0_	3.40	0.25	6.30	13.48
	a_cap,cap_	0.66	0.38	0.96	2.61
	a_cap,pcod_	0.34	−0.16	0.88	1.58
	a_cap,cod_	−0.51	−1.05	0.04	−4.20
	a_cap,herring_	−0.33	−0.70	0.07	−0.38
	a_cap,st_	0.20	−0.21	0.61	0.20
	a_cap,F_	−0.27	−0.68	0.12	−0.27
polar cod	a_pcod,0_	2.52	−1.17	6.53	11.78
	a_pcod,pcod_	0.45	−0.04	0.89	2.12
	a_pcod,cap_	0.01	−0.34	0.35	0.03
	a_pcod,cod_	0.07	−0.41	0.54	0.60
	a_pcod,herring_	0.17	−0.32	0.63	0.20
	a_pcod,ice_	0.26	−0.12	0.69	0.26
	a_pcod,*F*_	0.18	−0.21	0.54	0.18

For cod, the model shows a positive effect of two of the interspecific terms (capelin and polar cod). Temperature and herring abundance have no effect. The strongest effect on cod is the positive effect of the intraspecific term followed by the effect of the polar cod abundance, capelin abundance and the negative effect of F (table 1 and figure 2).

For capelin, the model shows a positive effect of polar cod and a negative effect of both cod and herring. In addition, capelin is positively affected by temperature. This means that the scenario of decreased sea ice reduction should positively affect capelin through the concomitant increase of sea temperature. The strongest effect on capelin is the negative effect of NEA cod abundance followed by the positive effect of the intraspecific term and the polar cod abundance, and the negative effect of herring abundance and F (table 1 and figure 2).

For polar cod, the model shows no effect of capelin and NEA cod but a positive effect of herring abundance. Polar cod is positively affected by the mean winter sea ice index (Ice). The latter means that the scenario of sea ice reduction should negatively affect polar cod. The strongest effect on polar cod is the positive effect of the intraspecific term followed by the positive effect of sea ice extent (table 1 and figure 2).

The decrease of the mean winter sea ice index in the Barents Sea (under the 50% observed during the studied period, see electronic supplementary material, figure S2A*,* and the corresponding increase of sea temperature, see electronic supplementary material, figure S2B) affects the different species by increasing the variability around the median (red dashed area in figure 3*a–c*, note the log scale) comparatively to the unperturbed model output (in blue). For NEA cod (figure 3*a*), under the low sea ice and high sea temperature scenario, the simulation indicates a decrease in abundance. A level of decrease in abundance is also visible after 2005, at least for some years, when the scenario affects the sea ice extent the least. For polar cod, the simulation indicates a decrease in abundance (figure 3*c*). For capelin (figure 3*b*), the simulation indicates an increased abundance, particularly before 2005 when the scenario strongly affects the sea ice extent.

Figure 3*d–f* focuses on the comparison of median abundances obtained by the scenario with the ones from the unperturbed models. For cod (figure 3*d*), the scenario of reduced sea ice and increased temperature leads to a decrease in abundance (higher decrease for higher abundance). If we assume linear regressions with fixed intercept going through the point of origin, we can obtain a slope for the period before 2005 (orange triangles) and for the period after 2005 (blue dots) for figure 3*d–f*. The difference of slopes is approximately 0.47 for capelin while it is approximately 0.15 for NEA cod and approximately 0.08 for polar cod. These results show a change in the intensity of the effect of sea ice reduction: strong before 2005 and weaker after 2005. For capelin (figure 3*e*), the scenario leads to an increased abundance while it leads to a decreased abundance for polar cod (figure 3*f*). For capelin, the difference indicates a strong effect of the scenario before 2005 (orange triangles) but less afterwards (blue dots in figure 3*e*). The sea ice reduction seems to only weakly benefit capelin abundance in the years after 2005. By contrast, with a low difference of slopes, the sea ice reduction negatively affects NEA cod and polar cod also after 2005, indicating some kind of propagation of the effect (figure 3*d,f*). Note that the median trajectories of the scenario stayed similar to the ones of the unperturbed model (electronic supplementary material, figure S5).

## 4. Discussion

Since the 1980s, sea ice cover in the Barents Sea has decreased concomitantly with temperature increase, both changes strongly affecting the ecosystem [4,7,44]. Using a state-space model that combines long-term population time series from scientific surveys with environmental variables, we describe the dynamics of three key species in this boreal-Arctic sea. By applying a scenario with sea ice reduction and sea temperature increase to our model, we demonstrate that climate change is expected to limit the population of an ice-associated Arctic species, the polar cod, and to provide an advantage to a boreal one, the capelin, supporting our initial hypothesis. By contrast, our scenario indicates that a major boreal predator, the NEA cod, seems not to benefit from the climate warming-associated sea ice loss and sea temperature increase.

The originality of our approach is a model with the concomitant effect of three interacting species, i.e., modelling inter- and intraspecies dynamics for addressing the biological impacts of changes in sea ice. In addition, our scenario kept the process error of the unperturbed model to keep the same three-way process dynamics that happened between the three species.

For polar cod, our scenario indicates that the reduction of sea ice leads to a stable (or slightly decreased) population abundance. This is in line with the conclusions of other recent studies [12,13,45]. Likewise, we found that the polar cod abundance is mainly associated with sea ice extent (abiotic factor) and that predator abundance (biotic factor) has relatively little effect (table 1 and figure 2).

For NEA cod, our scenario indicates that the increase in sea temperature in the Barents Sea is not associated with an increase but rather a decreasing abundance. This is counterintuitive since it was shown that NEA cod biomass is positively correlated to temperature [46] and a warming of the Barents Sea is expected to benefit the cod [47]. A positive relationship was shown between temperature and growth for the juvenile NEA cod in the Barents Sea [48], and between temperature and survival [49]. Our result is, however, in line with a reduction of the NEA cod population predicted in recent studies [50], shown in recent assessment results [51] and projected under the most commonly used emission scenario (SSP2-4.5, [52]). Note that the temperature effect is weak compared with the effect of other species, suggesting another process rather than a direct environmental effect. Here, our analysis shows that the documented negative effect of climate change on the NEA cod population [50] is mediated through changes in trophic interaction (prey availability and competition). For instance, the polar cod is the second most influential variable for NEA cod, and the polar cod population is going down in the low sea ice scenario. Our model suggests that the sea ice reduction negatively affects the polar cod abundance and may have a propagating effect (a chain effect) on NEA cod and on from here to capelin. Although while polar cod is not highly represented in the NEA cod diet in terms of mass ingested, it is the third prey item in terms of number [20]. Polar cod could affect NEA cod by being a major prey at certain times of the year. However, it is also possible that the effect on the NEA cod dynamics is caused by the polar cod being a proxy for other processes, such as environmental conditions experienced during the early life of cod or as a general proxy for the Barents Sea ecosystem health.

For capelin, our scenario indicates that the increase in sea temperature in the Barents Sea will lead to an increase in abundance (table 1). This contrasts with a recent study that projected a decrease in capelin productivity as temperature increases, using the SSP2-4.5 emission scenario [52], highlighting the importance of accounting for interactions in projection models. The positive effect of temperature on capelin abundance was expected. Previous studies have shown a positive relationship between sea temperature and the growth in length of capelin in the Barents Sea [53], as well as between sea temperature and capelin survival [54].

However, the strongest effect in our model is the negative effect of NEA cod abundance on capelin abundance followed by the positive effect of polar cod. The negative contribution of NEA cod abundance could be related to the temperature contribution (electronic supplementary material, figure S6). This may indicate that the climate effect on the capelin population could be an indirect effect through species interactions [54,55]. The polar cod affects NEA cod, which in turn affects capelin. The positive effect of polar cod on capelin indicates a covariation or release of predation pressure when polar cod is abundant. The latter, linked to the low effect of capelin on polar cod, may indicate that capelin is not a mitigation factor for the predation effect on the polar cod dynamics as previously assumed [12,18,56]. However, we cannot exclude such a mechanism since we did not account for other predators in our model. Nevertheless, the low process error indicates that this may not be a major mechanism (electronic supplementary material, figure S4).

Our model is relatively simple concerning the number of species included. While our model describes the data well with high explanatory power (electronic supplementary material, figure S4), several other variables could have also been considered. In particular, we could have included more prey components in our model, e.g., copepods, krill and amphipods (particularly for capelin, [55,57]). Likewise, we could have considered more predators beyond NEA cod and herring. For instance, marine mammals and seabirds are known to prey on NEA cod, polar cod and capelin [58,59]. However, the focus of our study was on the interaction between cod, capelin and polar cod in the northern Barents Sea, and it would have been difficult to model the other variables dynamically due to limitations in time series length and data availability. For instance, we did not model the herring population as we did for the NEA cod, polar cod and capelin, i.e. herring abundance does not vary with changes in environmental conditions such as sea temperature and species interactions. Consequently, the herring abundance time series was directly used as a covariate and did not change when used in the hindcast scenario and thus should have little effect on the scenario outcome for NEA cod, polar cod and capelin. However, the magnitude of the process error variance was low, suggesting that the potential influence of all aforementioned variables was relatively weak (electronic supplementary material, figure S4).

Our results are in line with other studies showing that sea ice extent affects the food chain in the Barents Sea [55]. For our scenario, the sea ice is only reduced to the level observed in the last 12 years of the studied time series (electronic supplementary material, figure S2). Following the projections of sea ice reduction for the next decades in the Barents Sea [1], our scenario is quite conservative and never leads to a sea ice-free Barents Sea as expected by climate models for the next decades [60]. This suggests that, relative to our scenario, the effect of climate change may lead to a stronger reduction of the polar cod population, eventually being replaced by capelin as a key forage fish species in the Arctic area of the Barents Sea ecosystem. The survival of polar cod eggs [14] and young age classes [12,61] are strongly associated with changes in sea ice. Knowing the sensitivity of fish population dynamics to young age classes’ mass mortality events [62,63], sea ice reduction is making the polar cod potentially more prone to population collapses. The reduction of the polar cod population size in a warmer climate will affect the transfer of energy along the food chain toward Arctic top predators if its central role within the Arctic food web is not taken over by boreal species such as the capelin. Our results suggest that this could be the case in the Barents Sea. However, important plankton prey species for capelin, such as *Calanus finmarchicus*, are also affected by the borealization, and the capelin population will need to adjust to an altered plankton bloom phenology and distribution with potential spatio-temporal mismatch [64,65]. The brief warm period of the 1930s [66] led to the northward expansion of boreal fish (such as cod, haddock (*Melanogrammus aeglefinus*) and herring (*C. harengus*)) in the Barents Sea and a northward retreat of colder water species (such as polar cod) [67]. This expansion was reversed during the cold period in the 1970s that followed. However, the food web did not completely revert to its previous state, as some alterations in the trophic structure persisted after the return of the cold period [24].

## 5. Conclusion

In a time of rapid ‘borealization’ of the Arctic seas causing extensive population redistribution and reordering of the food webs, it is imperative to understand how sea ice reduction will affect the Arctic food web and the flow of energy between the different trophic levels [68]. The sea ice effect was previously shown to cascade through the food web through indirect and delayed effects [55]. Here, using a three-species model, we show that by only considering the trophic interactions at the upper level, we can simulate the expected outcome of climate change. Stopping the reduction of the sea ice extent and, consequently, its effects is not feasible, at least not in the short term. However, our results indicate that the consequences of climate change may be mediated through trophic interactions. For example, using an end-to-end ecosystem model, recent work has suggested that polar cod could benefit from increasing fishing mortality of cod [69]. Improving our insights into the factors that determine population interactions will enrich ecology and aid in proposing new adaptation and mitigation measures for climate change.

## Data Availability

Northeast Arctic cod and capelin data are freely available in the report of the Arctic Fisheries Working Group (AFWG) 2019 of the International Council for the Exploration of the Sea [33]. Polar cod data are freely available in the survey reports from the joint Norwegian/Russian ecosystem survey in the Barents Sea at [34,35]. Herring in the Barents Sea is freely available at [36]. Kola sea temperature from the Polar branch of the Russian Federal Institute of Fisheries and Oceanography is available at [70]. See electronic supplementary material, table S1 [71]. The sea ice data are freely available at [72].

## References

[1] Pan R, Shu Q, Song Z, Wang S, He Y, Qiao F. 2023 Simulations and projections of winter sea ice in the Barents Sea by CMIP6 climate models. Adv. Atmos. Sci. **40**, 2318–2330. (10.1007/s00376-023-2235-2)

[2] Lind S, Ingvaldsen RB, Furevik T. 2018 Arctic warming hotspot in the northern Barents Sea linked to declining sea-ice import. Nat. Clim. Chang. **8**, 634–639. (10.1038/s41558-018-0205-y)

[3] Onarheim IH, Årthun M. 2017 Toward an ice‐free Barents Sea. Geophys. Res. Lett. **44**, 8387–8395. (10.1002/2017GL074304)

[4] Fossheim M, Primicerio R, Johannesen E, Ingvaldsen RB, Aschan MM, Dolgov AV. 2015 Recent warming leads to a rapid borealization of fish communities in the Arctic. Nat. Clim. Chang. **5**, 673–677. (10.1038/nclimate2647)

[5] Frainer A, Primicerio R, Kortsch S, Aune M, Dolgov AV, Fossheim M, Aschan MM. 2017 Climate-driven changes in functional biogeography of Arctic marine fish communities. Proc. Natl Acad. Sci. USA **114**, 12202–12207. (10.1073/pnas.1706080114) 29087943 PMC5699037

[6] Stige LC, Kvile KØ. 2017 Climate warming drives large-scale changes in ecosystem function. Proc. Natl Acad. Sci. USA **114**, 12100–12102. (10.1073/pnas.1717090114) 29093162 PMC5699100

[7] Pecuchet L, Blanchet MA, Frainer A, Husson B, Jørgensen LL, Kortsch S, Primicerio R. 2020 Novel feeding interactions amplify the impact of species redistribution on an Arctic food web. Glob. Chang. Biol. **26**, 4894–4906. (10.1111/gcb.15196) 32479687

[8] von Biela VR, Laske SM, Stanek AE, Brown RJ, Dunton KH. 2023 Borealization of nearshore fishes on an interior Arctic shelf over multiple decades. Glob. Chang. Biol. **29**, 1822–1838. (10.1111/gcb.16576) 36565055

[9] Gjøsæter H. 1998 The population biology and exploitation of capelin (Mallotus villosus) in the Barents Sea. Sarsia **83**, 453–496. (10.1080/00364827.1998.10420445)

[10] Fall J, Ciannelli L, Skaret G, Johannesen E. 2018 Seasonal dynamics of spatial distributions and overlap between Northeast Arctic cod (Gadus morhua) and capelin (Mallotus villosus) in the Barents Sea. PLoS One **13**, e0205921. (10.1371/journal.pone.0205921) 30325964 PMC6191152

[11] Johannesen E, Yoccoz NG, Tveraa T, Shackell NL, Ellingsen KE, Dolgov AV, Frank KT. 2020 Resource-driven colonization by cod in a high Arctic food web. Ecol. Evol. **10**, 14272–14281. (10.1002/ece3.7025) 33391714 PMC7771159

[12] Dupont N, Durant JM, Gjøsæter H, Langangen Ø, Stige LC. 2021 Effects of sea ice cover, temperature and predation on the stock dynamics of the key Arctic fish species polar cod Boreogadus saida. Mar. Ecol. Prog. Ser. **677**, 141–159. (10.3354/meps13878)

[13] Geoffroy M *et al*. 2023 The circumpolar impacts of climate change and anthropogenic stressors on Arctic cod (Boreogadus saida) and its ecosystem. Elem. Sci. Anth. **11**. (10.1525/elementa.2022.00097)

[14] Hop H, Gjøsæter H. 2013 Polar cod (Boreogadus saida) and capelin (Mallotus villosus) as key species in marine food webs of the Arctic and the Barents Sea. Mar. Biol. Res. **9**, 878–894. (10.1080/17451000.2013.775458)

[15] Frank KT, Petrie B, Choi JS, Leggett WC. 2005 Trophic cascades in a formerly cod-dominated ecosystem. Science **308**, 1621–1623. (10.1126/science.1113075) 15947186

[16] Van Leeuwen A, De Roos AM, Persson L. 2008 How cod shapes its world. J. Sea Res. **60**, 89–104. (10.1016/j.seares.2008.02.008)

[17] Johannesen E, Lindstrøm U, Michalsen K, Skern-Mauritzen M, Fauchald P, Bogstad B, Dolgov AV. 2012 Feeding in a heterogeneous environment: spatial dynamics in summer foraging Barents Sea cod. Mar. Ecol. Prog. Ser. **458**, 181–197. (10.3354/meps09818)

[18] Orlova EL, Dolgov AV, Rudneva GB, Oganin IA, Konstantinova LL. 2009 Trophic relations of capelin Mallotus villosus and polar cod Boreogadus saida in the Barents Sea as a factor of impact on the ecosystem. Deep Sea Res. II Top. Stud. Oceanogr. **56**, 2054–2067. (10.1016/j.dsr2.2008.11.016)

[19] Eriksen E *et al*. 2019 The working group on the integrated assessments of the barents sea (WGIBAR). In ICES sci rep (eds E Eriksen, A Filin), p. 157. Copenhagen, Denmark: International Council for the Exploration of the Sea.

[20] Holt RE, Bogstad B, Durant JM, Dolgov AV, Ottersen G. 2019 Barents Sea cod (Gadus morhua) diet composition: long-term interannual, seasonal, and ontogenetic patterns. ICES J. Mar. Sci. **76**, 1641–1652. (10.1093/icesjms/fsz082)

[21] Townhill B, Holt RE, Bogstad B, Durant JM, Pinnegar JK, Dolgov AV, Yaragina NA, Johannesen E, Ottersen G. 2021 Diets of the Barents Sea cod (Gadus morhua) from the 1930s to 2018. Earth Syst. Sci. Data **13**, 1361–1370. (10.5194/essd-13-1361-2021)

[22] Hjermann DØ, Ottersen G, Stenseth NC. 2004 Competition among fishermen and fish causes the collapse of Barents Sea capelin. Proc. Natl Acad. Sci. USA **101**, 11679–11684. (10.1073/pnas.0402904101) 15286282 PMC511038

[23] Gjøsæter H, Bogstad B. 1998 Effects of the presence of herring (Clupea harengus) on the stock-recruitment relationship of Barents Sea capelin (Mallotus villosus). Fish. Res. **38**, 57–71. (10.1016/S0165-7836(98)00114-3)

[24] Durant JM, Aarvold L, Langangen Ø. 2021 Stock collapse and its effect on species interactions: cod and herring in the Norwegian-Barents Seas system as an example. Ecol. Evol. **11**, 16993–17004. (10.1002/ece3.8336) 34938487 PMC8668721

[25] Durant JM, Hjermann DØ. 2017 Age-structure, harvesting and climate effects on population growth of Arcto-boreal fish stocks. Mar. Ecol. Prog. Ser. **577**, 177–188. (10.3354/meps12210)

[26] Hjermann DØ, Bogstad B, Eikeset AM, Ottersen G, Gjøsæter H, Stenseth NC. 2007 Food web dynamics affect Northeast Arctic cod recruitment. Proc. R. Soc. B **274**, 661–669. (10.1098/rspb.2006.0069) PMC2197215 17254990

[27] Tytler P, Calow P. 1985 Fish energetics, new perspectives, p. 349. Springer Dordrecht. (10.1007/978-94-011-7918-8)

[28] Butzin M, Pörtner HO. 2016 Thermal growth potential of Atlantic cod by the end of the 21st century. Glob. Chang. Biol. **22**, 4162–4168. (10.1111/gcb.13375) 27378512

[29] Durant JM, Ono K, Langangen Ø. 2022 Empirical evidence of nonlinearity in bottom up effect in a marine predator-prey system. Biol. Lett. **18**, 20220309. (10.1098/rsbl.2022.0309) 36321432 PMC9627449

[30] Durant JM, Ono K, Stenseth NC, Langangen Ø. 2020 Nonlinearity in interspecific interactions in response to climate change: cod and haddock as an example. Glob. Chang. Biol. **26**, 5554–5563. (10.1111/gcb.15264) 32623765

[31] Ono K, Langangen Ø, Stenseth NC. 2019 Improving risk assessments in conservation ecology. Nat. Commun. **10**, 2836. (10.1038/s41467-019-10700-4) 31249288 PMC6597725

[32] Bakketeig IE, Hauge M, Kvamme C, Sunnset BH, Toft KØ. 2016 Havforskningsrapporten 2016. Fisken Havet **1**, 99.

[33] ICES. 2019 Arctic fisheries working group (AFWG). ICES. Sci. Rep. **01**, 934. (10.17895/ices.pub.5292)

[34] van der Meeren GI, Prozorkevich D. 2021 Survey report from the joint Norwegian/Russian ecosystem survey in the Barents Sea and adjacent waters, August-November 2020. In IMR/PINRO joint report series (eds GI van der Meeren, D Prozorkevich), p. 123.

[35] Protzorkevich D, van der Meeren GI. 2020 Survey report from the joint Norwegian/Russian ecosystem survey in the Barents Sea and adjacent waters, August-October 2019. In IMR/PINRO joint report series (eds D Protzorkevich, GI van der Meeren), p. 93.

[36] Eriksen E. 2022 The Barents Sea ecosystem time series 1980–2019. (10.21335/NMDC-1069717541)

[37] ICES. 2021 Arctic fisheries working group (AFWG). ICES. Sci. Rep. **3**, 817. (10.17895/ices.pub.8196)

[38] Langangen Ø, Ohlberger J, Stige LC, Durant JM, Ravagnan E, Stenseth NC, Hjermann DØ. 2017 Cascading effects of mass mortality events in Arctic marine communities. Glob. Chang. Biol. **23**, 283–292. (10.1111/gcb.13344) 27151543

[39] Ives AR, Dennis B, Cottingham KL, Carpenter SR. 2003 Estimating community stability and ecological interactions from time-series data. Ecol. Monogr. **73**, 301–330. (10.1890/0012-9615(2003)073[0301:ECSAEI]2.0.CO;2)

[40] Stan Development Team. 2021 RStan: the R interface to Stan. R package version 2.21.3.

[41] Ono K, Punt AE, Rivot E. 2012 Model performance analysis for Bayesian biomass dynamics models using bias, precision and reliability metrics. Fish. Res. **125–126**, 173–183. (10.1016/j.fishres.2012.02.022)

[42] Vehtari A, Gelman A, Simpson D, Carpenter B, Bürkner PC. 2021 Rank-normalization, folding, and localization: an improved rhat for assessing convergence of MCMC (with discussion). Bayesian Anal. **16**, 667–718. (10.1214/20-BA1221)

[43] R Core Team. 2022 R: A language and environment for statistical computing. R Foundation for Statistical Computing, Vienna, Austria. See http://www.R-project.org.

[44] Eriksen E, Skjoldal HR, Gjøsæter H, Primicerio R. 2017 Spatial and temporal changes in the Barents Sea pelagic compartment during the recent warming. Prog. Oceanogr. **151**, 206–226. (10.1016/j.pocean.2016.12.009)

[45] Gjøsæter H, Huserbråten M, Vikebø F, Eriksen E. 2020 Key processes regulating the early life history of Barents Sea polar cod. Polar Biol. **43**, 1015–1027. (10.1007/s00300-020-02656-9)

[46] Koul V, Sguotti C, Årthun M, Brune S, Düsterhus A, Bogstad B, Ottersen G, Baehr J, Schrum C. 2021 Skilful prediction of cod stocks in the North and Barents Sea a decade in advance. Commun. Earth Environ. **2**, 140. (10.1038/s43247-021-00207-6)

[47] Kjesbu OS, Bogstad B, Devine JA, Gjøsæter H, Howell D, Ingvaldsen RB, Nash RDM, Skjæraasen JE. 2014 Synergies between climate and management for Atlantic cod fisheries at high latitudes. Proc. Natl Acad. Sci. USA **111**, 3478–3483. (10.1073/pnas.1316342111) 24550465 PMC3948268

[48] Brander KM. 1995 The effect of temperature on growth of Atlantic cod (Gadus morhua L.). ICES J. Mar. Sci. **52**, 1–10. (10.1016/1054-3139(95)80010-7)

[49] Opdal AF, Vikebø FB, Fiksen Ø. 2011 Parental migration, climate and thermal exposure of larvae: spawning in southern regions gives Northeast Arctic cod a warm start. Mar. Ecol. Prog. Ser. **439**, 255–262. (10.3354/meps09335)

[50] Årthun M, Bogstad B, Daewel U, Keenlyside NS, Sandø AB, Schrum C, Ottersen G. 2018 Climate based multi-year predictions of the Barents Sea cod stock. PLoS One **13**, e0206319. (10.1371/journal.pone.0206319) 30356300 PMC6200261

[51] ICES. 2023 Arctic fisheries working group (AFWG; outputs from 2022 meeting). ICES. Sci. Rep. **5**, 507. (10.17895/ices.pub.20012675)

[52] Ma S, Huse G, Ono K, Nash RDM, Vølstad JH, Kjesbu OS. 2024 Northeast Atlantic fish stock productivity hindcasts and forecasts from a Bayesian framework reveal pronounced climate‐induced dynamics. Fish Fish. **25**, 686–710. (10.1111/faf.12833)

[53] Gjøsæter H, Loeng H. 1987 Growth of the Barents Sea capelin, Mallotus villosus, in relation to climate. Environ. Biol. Fishes **20**, 293–300. (10.1007/BF00005300)

[54] Hjermann DØ, Stenseth NC, Ottersen G. 2004 Indirect climatic forcing of the Barents Sea capelin: a cohort effect. Mar. Ecol. Prog. Ser. **273**, 229–238. (10.3354/meps273229)

[55] Stige LC, Ono K, Eriksen E, Dalpadado P. 2019 Direct and indirect effects of sea ice cover on major zooplankton groups and planktivorous fishes in the Barents Sea. ICES J. Mar. Sci. **76**, 24–36. (10.1093/icesjms/fsz063)

[56] Nilssen KT, Pedersen OP, Folkow LP, Haug T. 2000 Food consumption estimates of Barents Sea harp seals. NAMMCO. Sci. Publ. **2**, 9–27. (10.7557/3.2968)

[57] Stige LC, Kvile KØ, Bogstad B, Langangen Ø. 2018 Predator-prey interactions cause apparent competition between marine zooplankton groups. Ecology **99**, 632–641. (10.1002/ecy.2126) 29281755

[58] Jakobsen T, Ozhigin VK. 2011 The Barents Sea: ecosystem, resources, management. Half a century of Russian - Norwegian cooperation, p. 825. Trondheim, Norway: Tapir Academic Press.

[59] Durant JM, Skern-Mauritzen M, Krasnov YV, Nikolaeva NG, Lindstrøm U, Dolgov A. 2014 Temporal dynamics of top predators interactions in the Barents Sea. PLoS One **9**, e110933. (10.1371/journal.pone.0110933) 25365430 PMC4218717

[60] Årthun M, Onarheim IH, Dörr J, Eldevik T. 2021 The seasonal and regional transition to an ice‐free Arctic. Geophys. Res. Lett. **48**, e2020GL090825. (10.1029/2020GL090825)

[61] Huserbråten MBO, Eriksen E, Gjøsæter H, Vikebø F. 2019 Polar cod in jeopardy under the retreating Arctic sea ice. Commun. Biol. **2**, 407. (10.1038/s42003-019-0649-2) 31728418 PMC6838109

[62] Langangen Ø, Ohlberger J, Stige LC, Patin R, Buttay L, Stenseth NC, Ono K, Durant JM. 2023 Effects of early life mass mortality events on fish populations. Fish Fish. **24**, 176–186. (10.1111/faf.12718)

[63] Langangen Ø, Durant JM. 2024 Persistence of fish populations to longer, more intense, and more frequent mass mortality events. Glob. Chang. Biol. **30**, e17251. (10.1111/gcb.17251) 38519869

[64] Skaret G, Dalpadado P, Hjøllo SS, Skogen MD, Strand E. 2014 Calanus finmarchicus abundance, production and population dynamics in the Barents Sea in a future climate. Prog. Oceanogr. **125**, 26–39. (10.1016/j.pocean.2014.04.008)

[65] Durant JM, Hjermann DØ, Ottersen G, Stenseth NC. 2007 Climate and the match or mismatch between predator requirements and resource availability. Clim. Res. **33**, 271–283. (10.3354/cr033271)

[66] Bengtsson L, Semenov VA, Johannessen OM. 2004 The early twentieth-century warming in the Arctic—a possible mechanism. J. Clim. **17**, 4045–4057. (10.1175/1520-0442(2004)017<4045:TETWIT>2.0.CO;2)

[67] Drinkwater KF. 2006 The regime shift of the 1920s and 1930s in the North Atlantic. Prog. Oceanogr. **68**, 134–151. (10.1016/j.pocean.2006.02.011)

[68] Coyle KO, Eisner LB, Mueter FJ, Pinchuk AI, Janout MA, Cieciel KD, Farley EV, Andrews AG. 2011 Climate change in the southeastern Bering Sea: impacts on pollock stocks and implications for the oscillating control hypothesis. Fish. Oceanogr. **20**, 139–156. (10.1111/j.1365-2419.2011.00574.x)

[69] Nilsen I, Hansen C, Kaplan I, Holmes E, Langangen Ø. 2022 Exploring the role of Northeast Atlantic cod in the Barents Sea food web using a multi‐model approach. Fish Fish. **23**, 1083–1098. (10.1111/faf.12671)

[70] Tereschenko VV. 1996 Seasonal and year-to-year variations of temperature and salinity along the Kola meridian transect. ICES CM C11 24.

[71] Durant J, Dupont N, Ono K, Langangen Ø. 2024 Data from: Interaction between three key species in the sea-ice reduced Arctic Barents Sea system. Figshare. (10.6084/m9.figshare.c.7477869)39378999

[72] OSI-SAF. 2024 Sea-ice index. See https://osi-saf.eumetsat.int/products/osi-420.

